# From code to care: Clinician and researcher perspectives on an optimal therapeutic web portal for acute myeloid leukemia

**DOI:** 10.1371/journal.pone.0302156

**Published:** 2024-04-18

**Authors:** Terese Knoppers, Cassandra E. Haley, Sarah Bouhouita-Guermech, Julie Hagan, Jacqueline Bradbury-Jost, Samuel Alarie, Marie Cosquer, Ma’n H. Zawati

**Affiliations:** Centre of Genomics and Policy, McGill University, Montreal, Quebec, Canada; Free University of Berlin, GERMANY

## Abstract

**Background:**

Acute myeloid leukemia (AML), a rapidly progressing cancer of the blood and bone marrow, is the most common and fatal type of adult leukemia. Therapeutic web portals have great potential to facilitate AML research advances and improve health outcomes by increasing the availability of data, the speed and reach of new knowledge, and the communication between researchers and clinicians in the field. However, there is a need for stakeholder research regarding their optimal features, utility, and implementation.

**Methods:**

To better understand stakeholder perspectives regarding an ideal pan-Canadian web portal for AML research, semi-structured qualitative interviews were conducted with 17 clinicians, researchers, and clinician-researchers. Interview guides were inspired by De Laat’s “fictive scripting”, a method where experts are presented with scenarios about a future technology and asked questions about its implementation. Content analysis relied on an iterative process using themes extracted from both existing scientific literature and the data.

**Results:**

Participants described potential benefits of an AML therapeutic portal including facilitating data-sharing, communication, and collaboration, and enhancing clinical trial matchmaking for patients, potentially based on their specific genomic profiles. There was enthusiasm about researcher, clinician, and clinician-researcher access, but some disagreement about the nature of potential patient access to the portal. Interviewees also discussed two key elements they believed to be vital to the uptake and thus success of a therapeutic AML web portal: credibility and user friendliness. Finally, sustainability, security and privacy concerns were also documented.

**Conclusions:**

This research adds to existing calls for digital platforms for researchers and clinicians to supplement extant modes of communication to streamline research and its dissemination, advance precision medicine, and ultimately improve patient prognosis and care. Findings are applicable to therapeutic web portals more generally, particularly in genomic and translational medicine, and will be of interest to portal end-users, developers, researchers, and policymakers.

## Introduction

Emerging genomics research is accelerating precision medicine in acute myeloid leukemia (AML), including new modalities for prognosis and treatment. As research advances, some teams are now using therapeutic web portals to share their research with other researchers, clinicians, and patients. A therapeutic web portal is an online platform designed to consolidate and share aggregate health research data, findings and information related to a medical condition or area of study [1,2]. The use of therapeutic web portals has potential to greatly facilitate AML research advances and improve health outcomes by increasing the availability of data, the speed and reach of new knowledge, and the communication between researchers and clinicians in the field.

AML is a rapidly progressing cancer of the blood and bone marrow. It is the most common and fatal type of leukemia among adults [3]. AML generally occurs in older people; the median age of incidence is 68 [4]. In Canada, approximately 1,100 people a year receive a new AML diagnosis [5] and the rate of incidence is rising [6]. While prognosis has improved over the past few decades, the five-year survival rate, with treatment, stands at 65% for childhood AML (<age 15) and 23% for adult AML [6]. A significant number of people do not respond to chemotherapy, the first line of treatment, or else relapse after an initial response [7]. As genetic subtype is the single most important prognostic factor for predicting treatment responses and health outcomes, precision medicine holds much promise in this area to help prolong and save lives [8]. Researchers are working to identify more types and frequencies of pathogenic variants involved, as well as different prognostic markers and therapeutic targets [9]. They aim to increase the efficacy of treatment for a wider range of patients via prognostic tests that can identify unresponsiveness to current therapies as well as novel precision therapies [10].

Therapeutic portals are distinct from patient portals, whose function is to house individual patient health information and facilitate clinical interaction and follow-up [1]. There is a substantial body of literature regarding patient portal availability, benefits, and optimization [11]. Patient portals empower patients to participate in their care and improve health outcomes by streamlining communication with care teams, providing resources and information [12] and offering follow-ups and tracking [13]. They encourage shared decision making and risk communication, helping patients to acquire and understand accessible personalized health information, including through the use of multimedia information [14]. Patient portals are increasingly popular in genomic medicine, and interest is growing in this area [15]. It is anticipated that innovations in user-friendly design as well as features of other mobile health applications will further drive engagement [16,17]. They have become valuable tools for the public to increase access to services, improve service delivery, and enable continuity of care [15].

In contrast to the growing interest in and uptake of patient portals, therapeutic portals remain less common and less established both in practice and in the literature [1]. Therapeutic portals open new avenues for research collaboration, dissemination, and progress. They are therapeutic in that researchers may use them towards further research and to stay current in their field, clinicians to inform patient care, and patients to gain knowledge regarding their diagnosis and course of treatment. They operate alongside conferences and publications to disseminate knowledge but are unique in their accessibility as online platforms. They also potentially enhance the role of the researcher in communication to stakeholders and of web portals in clinical decision-making and care. In the broader context of systemic gaps and lags between research findings and translation, therapeutic portals can be situated as part of a move towards learning healthcare systems in which research and therapy are mutually supportive [10]. As such use significantly expands the scope and application of web portals, research regarding their optimal features and utility is apropos. Further, much needs to be understood in terms of their implications for accessibility, efficiency and transparency of information, research communication, progress and clinical translation, patient empowerment, decision-making, and equity of access, and confidentiality and security of health data. Cancer genomics is a particularly rich area in which to explore the potential benefits, applications, and ethical considerations of therapeutic web portals, as the field is rapidly evolving and researchers, clinicians, and the public alike have significant interest in efficient and timely access to, and advancement of, research, knowledge, and care [2,18].

The present study is attached to a larger multi-institution biobank-based research project in Quebec working towards advances in precision therapy for AML. In previous contributions from our research team, we described the emergence of therapeutic portals and discussed associated legal, ethical, and policy considerations raised in existing literature and regulatory documents [1,2]. Alongside these theoretical contributions, it is essential to engage with the stakeholders for whom these portals are being designed. Recognizing the need for the perspectives of potential end-users active in AML research and care, we interviewed researchers, clinicians, and clinician-researchers regarding an ideal therapeutic web portal to advance precision medicine for AML. Questions centered around potential end-users, content, and applications of the ideal web portal as well as barriers and drivers of its success. Overall, the study was designed to better conceptualize and understand the views of clinical and research-based end-users, including their ideas and practical and ethical concerns, as user stakeholder considerations are important and instructive to the design, implementation, and adoption of therapeutic web portals. To the best of our knowledge, within the small but growing body of literature related to therapeutic portals, this exploratory qualitative study is the first of its kind. Findings will be of interest to portal end-users, developers, researchers, and policymakers. As such portals remain novel, relatively unestablished, and thus particularly open to innovation and possibility, ideas and issues raised can be invaluable to optimizing their potential.

## Methods

### Research design

This study employed a qualitative descriptive design, via semi-structured interviews and content analysis, to explore the perspectives of AML clinicians, researchers, and clinician-researchers on an optimal therapeutic web portal [19]. Qualitative research allows for thoughtful, in depth, nuanced exploration of areas where stakeholder views are relatively unknown [20]. Semi-structured interviews were chosen to produce data that both addresses researcher inquiries and allows novel participant-driven concerns and priorities to emerge outside the interview template [20]. In descriptive content analysis, coding takes place at semantic and contextual levels and results are presented in straightforward language and organized to best represent the data [19,21]. A descriptive approach was appropriate to summarize the perspectives of participants across the data set and because of the practical focus of the research aims. Ethical approval for the study was obtained from the Research Ethics Board of McGill University’s Faculty of Medicine (IRB Study Number A02-E13-19A). Methods and findings have been reviewed in light of the consolidated criteria for reporting qualitative research (COREQ) [22]. The COREQ checklist is available as a (S1 Appendix).

### Recruitment

The study population was healthcare providers and researchers who have substantive experience working with patients with AML and/or conducting research in AML, and who work in Canada. Recruitment occurred over email between 15 March 2019 and 4 January 2021 via a combination of purposive and convenience sampling. Potential interviewees were identified through the authors’ professional networks as well as through collaborations with professional associations such as the Canadian Association of Medical Oncologists (CAMO), the Canadian Association of Nurses in Oncology (CANO), the Canadian Association of Psychosocial Oncology (CAPO) and the Canadian Hematology Society (CHS). Additionally, AML researchers funded by a Canadian grant agency or the Leukemia & Lymphoma Society of Canada (LLS) since 2016 were approached for participation, sourced from data available on the Canadian Research Information System [23] and the LLS website for researchers [24] respectively. Potential interviewees were sent an invitation email that included information about the study as well as a consent form. Those who expressed interest were asked to sign and return the consent form and encouraged to contact the research team with any questions about the study. Interviews were then scheduled based on mutual availability.

### Data collection

S.A. and J.H. conducted semi-structured interviews using interview guides inspired by De Laat’s “fictive scripting” [25]. This method relies on presenting experts with scenarios about a future technology and asking questions about its implementation. The purpose of this exercise is to promote reflection and identify existing gaps and barriers as well as ways to overcome them [26]. After describing the potential implementation of the web portal for precision medicine in AML, questions focused on: (1) which end-users should be considered in planning and implementation; (2) the features they would deem to be ideal in the web portal and (3) barriers and facilitators towards success in implementation. By considering a variety of narratives and perspectives on the hypothetical implementation of the technology, future therapeutic web portal innovation may be tailored with end-users’ ideas and feedback in mind [25]. The interview guide is available as S2 Appendix.

Ultimately, 17 interviews were conducted with researchers and clinicians across Canada during 2019 and 2020. To maximize accessibility, understanding that our study population is busy and hard to reach, participants chose between phone and Zoom interviews [27]. All participants chose to interview by phone. Interviews lasted 30 to 45 minutes in length and were audio recorded with participant consent. Five interviews took place in French and 12 were conducted in English. To determine sample size, the research team applied Malterud et al.’s (2016) ‘informational power’ model which considers the information a sample holds along five mutually impactful dimensions: the breadth of the study aim, the specificity of the target sample population, whether the study applies an established theoretical framework, the quality of interview dialogue, and the analysis strategy [28]. The researchers considered the narrow study aim, specialized study population, focused in-depth interview discussions, and framework of exploratory descriptive cross-case content analysis to generate practical ideas and feedback from participants, and deemed the study’s informational power to be sufficient after 17 interviews, concluding data collection.

### Data analysis

Five members of the research team took part in data analysis. Transcription of interviews was outsourced to a professional agency with expertise in qualitative studies contracted with the authors’ research centre under a confidentiality agreement. J.H. and S.A. then analyzed the transcripts using NVivo 12 software. Content analysis was iterative, engaging themes extracted from both existing scientific literature and the data [21]. Intercoder reliability best practices, compatible with the interpretivist epistemological paradigm of qualitative research, were used [29]. The coding process involved collaboratively developing themes and subthemes through analysis of an initial set of seven transcripts, after which J.H. and S.A. met to discuss and refine the identified themes. Agreed-upon definitions were then assigned to each theme and sub-theme, and a codebook was created. J.H. and S.A. then traded off coding and validating roles on sets of two subsequent transcripts at a time, adding to and revising the codebook as was relevant until the data was saturated and no new themes were emerging from iterative coding. At this point the codebook was finalized. The codebook is available as S3 Appendix. As a final validation measure, T.K, C.E.H. and M.C. went back into the NVivo file and validated the coding under the themes and subthemes. Then T.K. reorganized and synthesized the coded categories into themes relevant and important to three areas of focus under the central research question, in preparation for presentation in a narrative format [21]. T.K., C.E.H. and J.B-J. collaborated on writing the Results sections. As the research concerned an area lacking qualitative data, the emphasis was on representing salient themes across participant interviews, rather than in depth explorations of a set of themes. In the final report, supporting quotes have been de-identified, cleaned of fillers and dysfluencies, and translated to English where relevant [30].

### Participants

**Fig 1:**
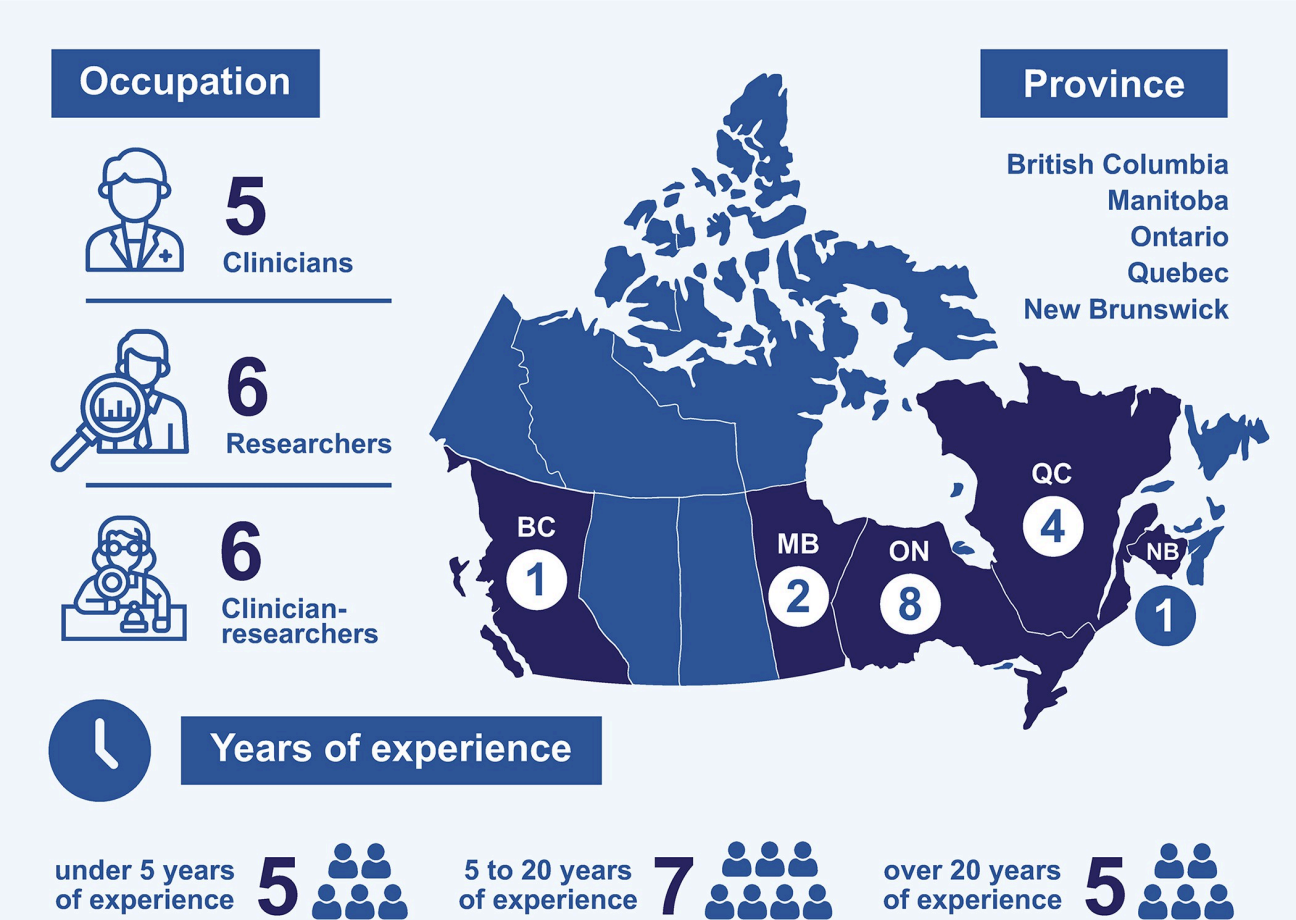
Aggregate participant demographics. Participants came from five provinces. Most interviewees worked in Quebec (4) and Ontario (8), but Manitoba (2), New Brunswick (1), and British Columbia (1) were also represented in the participant pool. Interviewees had a broad range of professional experience in their respective fields; from under one year to over four decades. Approximately one third of participants (5) had less than five years of professional experience, and one third reported more than 20 years’ professional experience (5), with the remaining participants falling within these boundaries (7). When asked if they were ‘a clinician, researcher or both’, five participants identified as clinicians (C), six identified as researchers (R), and the remaining six participants self-described as clinician-researchers (CR). It is important to note however, that these categories were not strictly discreet; clinician-researchers may have divided their time between the two disciplines roughly equally, but most interviewees reported exposure to both research and clinical work. The clinical backgrounds of interviewees included haematology, oncology, genetics, pharmacology, and immunology. Research areas included molecular biology, cancer biology, cell biology, genetics, gene therapy, tumorigenesis, immunology, preclinical drug trials, and translational research, across both model organisms (mice) and human cell lines.

As the size of the AML field in Canada presents an elevated risk of participant re-identifiability, only limited demographic information was gathered and results are presented in aggregate. Participant demographics are displayed in Fig 1.

## Results

**Fig 2:**
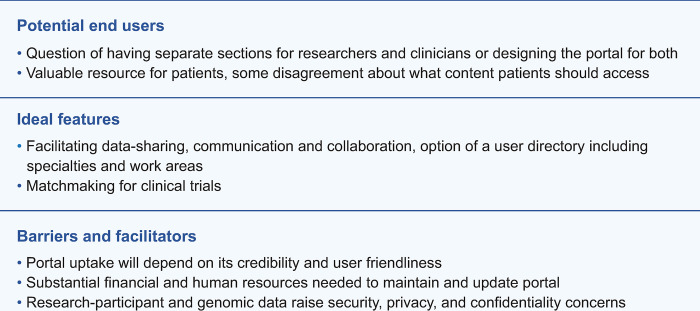
Summary of results: Clinician and researcher perspectives on an ideal AML web portal.

Themes and subthemes are presented below in tandem with the three main lines of inquiry of the interviews: (1) potential end users; (2) ideal features; and (3) barriers and facilitators. Notably, participants had different ideas regarding the nature of the data to be housed in an ideal therapeutic web portal; most discussed the value of centralized and sharable research data, but some also envisioned clinical data being present as well. Reflecting this blend of clinical and research data, the following analysis discusses both patient and research data as being potentially featured. Fig 2 gives an overview of findings.

### Potential end users

When asked which end-users should be considered in the planning and implementation of the web portal, it was universally assumed AML researchers and clinician-researchers would have access, and that there would be interest from clinicians, depending on the portal’s content. Interviewees made additional arguments for use by members of extended research and care teams but had more varied views regarding patient access.

#### Researchers and clinicians

Researchers and clinician-researchers expressed that they would utilize a therapeutic AML web portal housing genomic data and research to *“drive their research”* (R5). CR5: “*If you think of the pure basic research world*, *I mean this is a goldmine*.*”* Some researchers asserted that if the portal is to be used as a research tool, access should extend to all members of research teams: “*I’m sure you’re interviewing lots of PIs like myself*, *but the reality is…the people that are probably going to be accessing and using this portal at an in-the-trenches level*, *would be my post-docs and my graduate students”* (R2). It was further argued that students and fellows could also benefit from the portal as an educational resource supporting their training.

For the portal to be useful for and used by clinicians, interviewees stressed that it should also be geared towards them, as researchers and clinicians *“are used to navigating through computer systems and portals in different ways”* (CR5). CR1:

*It can be difficult for the clinician to understand the more fundamental elements of the research and vice versa in some cases when people don’t have both hats on*. *So*, *it’s interesting*, *but I think there needs to be a really important exercise where the language is common to both groups to really tie it all together*.

While some interviewees viewed a joint interface as an opportunity in of itself to enhance cross-disciplinary understanding and communication between clinicians and researchers, others pictured a web portal with specialized sections geared to research and clinician interests. C5:

*If you’re trying to put large genomic data in there*, *the question is*: *what’s really necessary from a research perspective and a clinical perspective*? *I think that from a clinical perspective…the portal should be clinically relevant to the patient’s case right now*. *I think there should be the ability to link to other genomic abnormalities but maybe not through the clinical part of the portal*, *through the research part of the portal*.

One concern was that clinicians may misinterpret the genomic data presented on the web portal, which could lead to faulty decision making: *“I don’t think we should take for granted that all of our people are hyper-knowledgeable in these areas*, *which are becoming more and more specialized”* (CR1); *“You have to be very*, *very careful with what’s preliminary and what’s proven”* (R4). Finally, several interviewees expressed that other professionals in extended care teams should have access to clinical data in the portal to facilitate information-sharing and ultimately improve care: *“I don’t see why that information shouldn’t be shared with all the people that are involved in the care of those patients*, *be it genetic counselors or nurses or other parties”* (R3).

#### Patients

While AML researchers and clinicians agreed patients should access the therapeutic portal, they had varied views on the type of content patients should access. There was general agreement that patients would benefit from a section of the portal designed to be accessible to the general public and with the interests of patients in mind: “*It could also be a portal of information for patients*. *To get information on the illness*, *things that are more popularized”* (CR3). Beyond the importance of having information on the portal relevant to patients however, some interviewees expressed that access to raw and/or specialized information should be restricted. Concerns revolved around understanding the utility of the information present as well as potential burdens and harm: “*Information needs to be communicated appropriately to not overwhelm the patient and to avoid any type of misunderstanding or misinterpretation of what’s going on out there”* (R3). One clinician-researcher voiced that patients may feel distressed by the information found in the portal and its potential implications for their prognosis: *“If I have a thirty year old who is p53 mutated*, *he is going to know that he has got a bad leukemia*, *but I’m not so sure that clicking*, *having a portal link and seeing exactly how bad it is*, *is super helpful”* (CR4). CR4 further noted that *“most hematologists don’t even understand what’s missing in our data”*, indicating patients untrained in this field may not gauge the limitations of uncontextualized data. Other respondents made autonomy, informed consent, and transparency arguments for patient access to all data on the portal, even if it is raw and complex. C4: *“If it’s going to become*, *like*, *the source of truth*, *then I think patients should have access to it…I think patients should have access to what’s state of the art and know what’s going on in the field and where things are going*.*”* This thread emphasized that people are often well-informed about their condition thanks to the internet and want to be more involved in decision-making regarding their care. C2: *“If they’re going to have truly informed consent about their care and want to make decisions*, *they need to have all of the information available*.*”*

### Ideal features

Participants expressed a variety of opinions regarding the optimal features and uses of a web portal, which fall into two main themes: 1) fostering data sharing, communication and collaboration, and 2) matchmaking for implementing clinical trials.

#### Data sharing, communication, and collaboration

Data sharing was viewed as a strong potential benefit of a therapeutic web portal. Participants described how sharing data could allow for centralization of clinical research and knowledge, reducing interprovincial silos and improving patient care. After all, as one participant described: “*We are part of one big*, *great country…look at it from a sick person’s perspective*. *Do they care that they’re a Quebec AML patient or a Manitoba AML patient*? *They’re just an AML patient and they want the best”* (R1). Centralizing demographic data on AML would enable researchers to leverage more complex data sets in evaluating potential treatments:

[*When you know] what the patient is clinically presenting with*, *what their lab characteristics are*, *what their performance status is*, *what treatment they got*, *how they responded*, *what their genetic make-up is*, *what the outcome was*, *what their second line therapy was and how long they responded for*, *you can then become much*, *much more sophisticated*. (R4)

Some interviewees also stressed the importance of aiming for an international scope for data-sharing: “*At some point if we don’t collaborate internationally*, *well*, *not much will move forward”* (C5).

AML researchers and clinicians believed that a web portal could foster greater communication and collaboration in the community, noting they are currently somewhat siloed due to geographic and professional barriers. For example, many clinicians expressed that connecting with colleagues is difficult due to high workloads:

*Leukemia is a place where communication is a bit hard because there are very few experts in Quebec*. *They are very busy*, *they are overworked… They are overwhelmed on all fronts… When I have a new acute leukemia [patient] I cannot call the eight centers to know if they have available protocols*. *It’s too labor intensive to do that*. (C1).

Several mentioned principally relying on their immediate professional networks: *“In general*, *people will ask people they know”* (CR3). To mitigate communication and collaboration barriers, participants envisioned that the web portal would have some kind of directory feature; “*Something up to date about who does what on that type of subject*. *What information is available in one place or another”* (C5). A web portal could thus *“enable different hematologists to contact each other… [and] to contact people with different open protocols in different hospitals”* (C5), as *“it is not information that is that easy to find”* (C1). It could further help create connections between the AML research and clinical communities: *“I think that kind of portal could greatly improve the relationship between medical doctors and leukemia experts”* (C1). A sentiment shared by C2:

*When I see new patients and I’m working them up with a diagnosis of AML*, *it might be helpful to be able to connect with the researcher and [ask questions such as] does this sample have to come from an aspirate*, *a biopsy*, *can it come from blood*?

However, increased communication and collaboration was not a foregone conclusion: “*It could isolate people more because the researchers have direct access and don’t need the clinician’s input*. *Or it could be the opposite*, *where in order to apply for data*, *you must go through a committee that brings people together”* (CR5).

#### Matchmaking

Finally, interviewees posited that a web portal could be very beneficial for connecting researchers to potential clinical trial participants, and for connecting patients to medical centres offering targeted clinical trials. This could reduce bureaucratic lag as clinicians source potential trials for their patients:

*If we had a portal that told us at the Canadian level there is a clinical trial targeting the patient that I saw in the clinic today*, *we could contact that clinician right away*, *I think that would help researchers a lot… It would make things a lot more efficient*. (CR3)

As well, several participants mentioned how a web portal could benefit patients with rare mutations by directing them to clinical trials interrogating their specific disease genotype. As R2 stated: “*the* a priori *identification of patients based on target is the basis of precision medicine”*. Therefore, a portal that leverages this data to identify trials could be a helpful tool for patients seeking to participate in research, and for clinicians to support recruitment into otherwise unknown trials. One researcher outlined how a web portal could support patient outcomes through providing previously inaccessible options for trial selection: “*if it helps them pick [a trial] in a very selective sense*, *you might even actually decrease adverse outcomes”* (R4).

Participants also mentioned that institutional barriers impeding patients from participating in clinical trials could be mitigated through using a web portal to share information: “*If people wanted to enrol patients into some new therapy that is not approved at their institution*, *they could look for that therapy and have contact information as to where their patients could perhaps join a trial”* (R5). Patients in different provinces, functionally unable to access clinical trials occurring in out-of-province health jurisdictions, could be connected to research happening across the country: “*As a society*, *I think that we need to support patients on all levels*, *federal*, *provincial and so these portals*, *I think*, *could break down those barriers to some extent*,*”* (R1). However, some interviewees were more skeptical, with the impracticalities of matchmaking between patients and geographically distant clinical trials poignantly described by one clinician:

*I think the problem is*, *number one*, *I’m in a smaller centre–and I’m in a prairie economic environment*. *So*, *the ability for my patients to travel to a study that is not open in my institution is usually pretty limited…the IMD studies that are happening with people that are trying new drugs are usually at these larger centers*, *and people have to be in the area to be able to access them*. *And my patient*, *who’s a farmer*, *who doesn’t have a lot of money*, *can’t afford to travel to Toronto*, *live in Toronto*, *and access that trial… So*, *what I would love to know is what financial supports are available for those patients to be able to participate*. *If there’s compensation or if there’s room for accommodation in the city* (CR5).

### Barriers and facilitators

Researchers and clinicians discussed credibility and user friendliness as two factors vital to the uptake and thus success of a therapeutic AML web portal. In terms of barriers, interviewees had concerns regarding the financial and human resources required to run and to maintain the portal as well as around security, privacy, and confidentiality.

#### Portal uptake

Credibility was the most important element of uptake for participants. They highlighted that the web portal should be reputable and trustworthy and named several related criteria. First, the portal should contain unbiased information and any affiliations should be reputable: *“I’m more likely to go to a portal that is set up by either a hospital*, *an institution*, *a foundation or something that is known*, *respected*, *and validated”* (CR2). Pharmaceutical companies came up for several participants in this context:

*It would probably be important for this site to be independent because if the site is funded*, *for example*, *by a pharmaceutical company*, *well*, *it’s going to be biased*, *or at least there’s going to be a perception of bias*. *Just in terms of credibility*. *It’s something that people are very sensitive about*, *with good reason* (CR3).

Second, the portal should contain as much relevant high-quality information as possible including links to other sources: “*They would have to trust that this was state of the art*. *That this was a true web of AML and getting everything that there was out there”* (C4). Interviewees thought people would be particularly interested in a portal that provided information that is not easily accessible elsewhere: “*There are some protocols…that are not published*. *We are not able to find them on the internet”* (C1). Third, clinicians and researchers emphasized the importance of the portal being kept up to date: *“Knowledge about cancer and genetics is moving very fast… If this portal is not updated regularly*, *it loses its interest”* (CR1). Finally, and most centrally, researchers and clinicians asserted that the web portal should drive medicine forward for AML: “*It’s all to go towards personalized medicine”* (R1).

Interviewees also spoke to the importance of user friendliness to portal uptake: *“People tend to gravitate to web sites and use portals that are very user friendly*. *When it becomes too complicated and too many things*, *too many colours*, *too many buttons*, *you don’t get engagement”* (R2). Key to usability was accessibility: finding the desired information efficiently and easily, design that minimizes a user’s cognitive load, and intuitive navigation (e.g. familiar icons and tools). This was in part because of how busy clinicians and researchers are. As one clinician-researcher put it:

*It has got to be a pretty simple interface and something that is easy to navigate… Not too many things nested within things where*, *you have to click this*, *then click ten other things before you find the info*. *Intuitive* (CR4).

Finally, in order to optimize use, interviewees further stipulated that the web portal should work well “*across multiple devices or browsers”* (C3).

#### Sustainability

Interviewees emphasized the necessity of financial resources both to set up the portal and to keep it up to date. As R1 succinctly described: *“It’s always money and politics*, *don’t you know*? *… Those are the two barriers to anything*.*”* CR4 clarified the economic barriers to data sharing within Canada: “*The main problem about our lack of data is that there’s no money for databases*. *It would be really cool to have a funded Canadian registry; that way we can generate better data and unify what we’re doing”*. There was further concern that access to the portal be free as participants were *“Not convinced [potential users] would be willing to pay for a subscription”* (CR1). Interviewees suggested the Canadian Institutes of Health Research (R1), Genome Canada (R4), Health Canada and provincial authorities (C5), and the Canadian Cancer Society (C2) and internal hospital research budgets (C2) as possible sources of funding. Long term, one researcher suggested applying to *“make it an agency…permanently funded by the government”*, although *“that’ll take a few years”* and *“you’d have to show proof of concept initially”* (R4).

The other major theme that arose in discussions of web portal sustainability was the human resources needed to power and maintain a web portal. Interviewees mentioned the necessity of having dedicated administrative and computer science support staff alongside data analysts: “*People will be maintaining this whole portal…there’s a significant amount of resources going into the data entry”* (R1), as well as *“a lot of people on the administrative side… [and] then people who could run the portal"* (C1). To keep the web portal updated, researchers would have to collaborate with “*the various major universities that do clinical trials in Canada and in the United-States*,*”* and perform “*quality checks and QI and QA assurances that the information going in is accurate and that the data is reliable*” (CR5). Interviewees also discussed maintenance of the web portal software and the necessity of a high download speed:

*All web portals are based on some sort of software…Software constantly has bugs…And somebody would have to maintain that…If you wanted to download sequencing information…The greater the depth and more annotation*, *the more cost and people resources required* (R2).

#### Security, privacy, and confidentiality

Participants expressed concern for the security and confidentiality of data: “*What happens if there’s a breach*? *What’s the process*?*”* (CR5). This was particularly true for those who envisioned health data being present, due to the inherent risks of sharing sensitive data.

Some interviewees posited that security might not necessitate too much concern, since patient data should not be housed on an external web portal: “*I don’t know that it needs to be secure because… I’m not sure that you would need individual patient’s data unless you were actively pursuing that study*, *which would mean you would have it anyways”* (R5). Those who envisioned clinical data being present were more concerned. As R1 emphasized: “*I think to be able to access that portal*, *you should have to sign confidentiality agreements that you are accessing primary patient material*.*”* Another researcher raised the issue of privacy law across provincial borders: “*If [data are] identified then the privacy laws become a bigger issue*. *Especially sharing the information across provinces”* (CR5). Many participants stressed the need to remove identifying features in any patient data added: *“Well*, *I think that the name of the patient or factors that identify the patient would not have to be entered in the platform…Confidentiality should be respected”* (C5); *You can limit data privacy [concerns] by anonymizing the data…You just give them a unique number and then you can share in an open web portal”* (R4). However, there was still concern for housing sequencing information––inherently identifiable data––on a web portal: *“the question there is if the data is de-identifiable*… *I would like the sequences kept in a place that is different… because of the identifiability that could happen for the entire dataset”* (CR5). CR5 also brought up return of results for patients: *“And then let’s say you do a panel that is above clinical standard of care and you find something on the patient*. *How do you identify the patient to return that information*?*”*

Finally, there was some dissonance between the need for data security and user friendliness: *“A username and a password is probably reasonable*. *You could piggyback that on some things that people have already signed up for”* (CR4). The concern was that too many security measures in portal access would *“discourage people from using it”* (C2).

## Discussion

Stakeholder interviews raised valuable ideas, concerns, and considerations regarding the optimal design, utility, and implementation of a therapeutic AML web portal. Significantly, participants in this study generally saw therapeutic web portals as having great potential towards the advancement of precision medicine. A central focus on advancing AML care factored into feedback regarding what should be on the portal, its applications, and even into questions of the design of the portal itself. This enthusiasm is significant in that this is stakeholder research; it evinces how valuable therapeutic portals could be for the field. While the purpose of this study was to initiate discussion regarding an ideal AML therapeutic web portal, findings are applicable to therapeutic web portals more generally, particularly in genomic and translational medicine. Below, we examine the practical and ethical implications of the stakeholder perspectives outlined above.

### Portal accessibility

Participant perspectives on accessibility highlight the value in being intentional around how and what information is communicated to different end user groups, with the end goal of maximizing portal relevance and utility. AML researchers and clinicians spoke to their shared needs and interests as well as practical considerations to be made regarding their different work and expertise. This included that some cross-disciplinary translation and contextualization may be necessary on the portal. For example, considerations around having either a shared interface or specialized sections for clinicians and researchers are not just technical; they have implications for usability, communication, and collaboration [2,31]. Interestingly, the participants who brought the idea for a shared interface forward were clinician-researchers with the vantage point of expertise in both camps. Indeed, while both options have advantages, a shared platform perhaps holds more potential towards bridging the work of researchers and clinicians. In terms of what content on the portal is available to patients, patient organizations and a growing body of research assert that patients are interested in raw genomic data as well as accessing scientific and clinical findings relevant to them as they are produced [32–35]. Authors have argued that data access empowers patients, encourages their further research participation, and that there are means to facilitate informed use [34,36,37]. Again, patients could benefit from contextualization of information present as well as a feature that summarizes research data in lay language. Finally, subthemes regarding end users and uptake evince a need to both minimize navigational barriers via web portal design [11,16,38] and to ensure end users are adequately prepared to effectively use the web portal [39–41]. The latter could be potentially achieved via embedded training and guidance [1,2].

### Data collection, sharing, and protection

Researchers and clinicians alike expressed optimism about the portal’s potential to help them share data across provinces and institutions, while raising bureaucratic, practical, and ethical challenges. Indeed, Canada continues to grapple with limitations in sharing health data between its provinces, due to current infrastructures and institutional barriers [42,43]. However, important work is being done to mitigate these limitations. Canada has implemented the Strategy for Patient-Oriented Research (SPOR) Evidence Alliance, an initiative designed to improve the sharing of data and protocols, validate information and definitions, and foster collaboration among stakeholders, attempting to overcome jurisdiction heterogeneity across provinces [44,45]. These existing platforms could set the tone for future therapeutic web portals as they encourage the standardization of protocols and concepts to facilitate effective data comparison and usage across provinces. Another inter-provincial consideration relates to the variation in AML prevalence from one province to another, including regional contexts. Ideally, the AML web portal would consider and gather relevant information pertaining to regional variation in AML prevalence. For example, Ontario has the highest group of people with AML in Canada, mainly clustered around five small industrial cities with significant environmental pollutants [46]. Considering provinces’ particularities is crucial to refine the information specific to AML research for respective populations, and ultimately, to better understand and combat AML. Finally, while one of the main stakeholder concerns was ensuring data privacy, there was dissensus on the best approach for data protection. Genomic sequencing is known to be challenging in anonymization [47,48] and risks are compounded for AML patients as it is a rare disease. Determining which information should be extracted and which should be retained poses a challenge. As evinced in the interviews, there is a balance to be made between safety, efficacy, and ease of use. Risks and benefits of the different options should be carefully weighed. However, many initiatives were taken to address these concerns, such as the Framework for responsible sharing of genomic and health-related data developed by the Global Alliance for Genomic & Health (GA4GH). This framework includes privacy protection statements indicating the importance of restricting re-identification of anonymized data and tailoring privacy measures in proportion to the nature of the data [49].

### Equity in access and application

Participant interviews also brought forward important equity considerations in portal implementation and uptake. Canada is geographically the second largest country in the world and its public health care system is managed and distributed at the provincial/territorial level. Different provinces and regions are differently resourced and generally more rural and remote areas will not provide all treatments, trials and tools, especially if they are newer, rarer or more specialized, as is the case for AML [35,50]. As one participant described: travel to major urban centers requires considerable time, energy, and financial resources. Thus, in Canada geographical and socioeconomic healthcare access inequities can be mutually compounding. Centralizing diagnosis and treatment information and making it widely available could help to ameliorate existing access inequities [1], particularly, as interviewees articulated, around clinical information that is more obscure or harder to find. Further, as AML incidence is regionally specific, a pan-Canadian resource is also an opportunity to better characterize its epidemiologic distribution (currently poorly understood) [46], compile locally relevant data, and encourage research translation attuned to local contexts and needs. Similarly, purposive recruitment is important to help ensure that the data is representative of the broader AML population in Canada and thus clinical developments will be equitably beneficial [2]. Overall, the larger issue raised in these considerations is the role and responsibility of the portal in assuring that it first, serves its purpose in advancing care, and second, does so equitably in access and application. Professional and regulatory guidance specific to therapeutic portals could be highly beneficial in this area [2].

### Quality and sustainability

Finally, researchers and clinicians identified that substantial human and financial resources would be required to run and maintain a therapeutic AML web portal. They argued that populating the web portal with high-quality, vetted data and resources, ensuring continuous updates to maintain functionality and accuracy, and implementing quality control measures on how data in web portals are sourced, organized, and used, would be all crucial to its credibility and effectiveness. Indeed, it is insufficient to have access to information if it cannot be used. These efforts would ensure that the web portal serves as an valuable resource, including in combatting misinformation often prevalent on the internet [51]. However, each stakeholder involved in the web portal lifecycle would have to contribute in an ethical manner to maintain these systems, starting with their design where developers’ practices would have to align with ethical obligations to demonstrate safety and efficacy [2]. It would also require researchers and clinicians to fulfill their responsibility and play an active role in adequately and ethically sharing and using data to further contribute to the advancement of science and healthcare [52]. Given that AML is highly heterogeneous at the molecular level, larger datasets with greater variables, will allow for the portal to be more effective. Finally, end users would have to exercise clear judgement before using accessible data and provide feedback when encountering potentially erroneous information or suggest features to improve these therapeutic portals [53].

Interviewees were also unclear as to how an AML web portal would be funded, raising questions about how costs will be assessed and funding sources (i.e., research institutions, public health). Defining and categorizing therapeutic web portals is challenging, as they do not fit into a particular group like research studies or commercial products, but rather something that is closer to a research engine storing a wide range of data. This lack of clarity highlights the difficulty of determining the type of funding. Furthermore, securing funding has historically been challenging for rare disease research [54]. At the same time, interviewees gave examples of existing applicable Canadian agency funds and others suggested creating or securing a permanent Canadian funding stream specific to the AML web portal. Notably, most participants wanted the portal to be funded publicly if possible. Indeed, the Canadian government has initiatives for rare disease research and treatment potentially applicable towards this end [55]. As the conceptualization of therapeutic web portals become more refined, the appropriate funding sources and the resources required will become clearer.

### Limitations

This research has some limitations. A qualitative approach was selected to explore participant perspectives. Any extrapolation should be done with caution and couched in the Canadian context for research and healthcare. Qualitative methods generate in depth rather than broadly generalizable findings [20]. The number of researchers involved in data analysis and our descriptive approach likely minimized researcher bias. Notably this method was employed instead of the more interpretive and latent meaning generation produced by thematic analysis. Similarly, our focus was on practical and ethical considerations, rather than critical or theoretical discussion. Given the purposive and convenience sampling method and that participation occurred on a self-selected bias, it is possible that interviewees were more knowledgeable and/or passionate about web portals than is average for AML researchers and clinicians in Canada. We cannot claim that our sample is representative of the broader population from which it draws. Finally, therapeutic and patient portals were sometimes spoken to interchangeably in participant responses, which had implications for concerns and considerations throughout both the Results and Discussion. While this could suggest that the distinction may not have been clear, it may also indicate that the two portal categories have overlap, and there are areas where the interests of patients, researchers, and clinicians intersect that could be explored further. A separate research project from our team will focus on patient perspectives regarding therapeutic web portals.

## Conclusions

This research adds to existing calls for digital platforms for researchers and clinicians to streamline research and its dissemination, advance precision medicine, and ultimately improve patient prognosis and care. Therapeutic web portals straddle the research and clinical worlds, thus holding potential to help foster the novel developments in translational research and clinical treatments that could occur with increased communication and collaboration between the two. In the case of AML, where prognosis is poor and many people are unresponsive to current therapies, such developments hold critical potential to prolong and save lives. Interviewing clinician-researchers, as well as clinicians and researchers with experience in both areas, was important in part because of their dual roles; they are uniquely positioned to give input on what kind of web portal could best further facilitate genomic AML research advances and translation into clinical practice [56]. The ideas and perspectives presented here represent a starting point for considerations in the envisioning and execution of ideal web portals for the advancement of genomic medicine. As therapeutic web portals are increasingly implemented, continuing to engage researchers and clinicians throughout the web portal lifecycle, will be essential to their success and sustainability. Further research into how they are used and applied by clinicians and researchers will be invaluable towards achieving their full promise.

## Supporting information

S1 Appendix COREQ checklist. (DOCX)

S2 Appendix Interview guide. (DOCX)

S3 Appendix Codebook. (DOCX)
