# Supplementary material for: From code to care: Clinician and researcher perspectives on an optimal therapeutic web portal for acute myeloid leukemia
Source: PLoS One. 2024 Apr 18;19(4):e0302156. doi: 10.1371/journal.pone.0302156 (PMC11025855; doi:10.1371/journal.pone.0302156)
Supplement: S2 Appendix — (DOCX) [file pone.0302156.s002.docx]

# Supporting information: Semi-structured interview guide

**Sociodemographic questions**

1. Please briefly describe your experience with AML.
2. How long have you been working in this field?
3. Do you consider yourself more as a clinician, as a researcher or as a clinician-researcher?

**Questions related to professional Internet and web portal use**

1. To what extent do you use the Internet to search for information as part of your professional practice?
2. Which types of information do you look for on the Internet in the course of your professional activities?
3. What devices do you use to access this information? (Computer, mobile devices)
4. Do you use, or have ever used, a web portal as part of your professional practice?

**Questions related to an ideal AML web portal**

1. Imagine that there was a web portal designed to feature research about acute myeloid leukemia. What sort of features would you want it to have?
2. Which types of information would you ideally like to have access to?
3. Who do you think would benefit from using this portal and why?
4. What might it do for healthcare providers and researchers?
5. Which relationships or professional networks should be established to help this web portal be successful?
6. What (and/or who) would determine the success of the web portal?
7. What would an ideal web portal need to be user-friendly? For example, visual aspects, navigation features, etc.
8. Ultimately, if you had to choose between the ease of use of the web portal or have access to more information and functionalities, which would you choose and why?
9. Imagine a world where there would be no barriers to the effective implementation of a web portal. Which barriers that currently exist, would not exist in this ideal world? For example, legal, technological, social, and cultural barriers.
10. What material, professional, technological, and other resources should be put in place to promote the optimal use of such a portal?
11. How do you think the updating of the web portal should be done? And, if so, who should be responsible for updating information?
12. In an ideal world, who would be the end-users of such a portal and how would they use it?
13. How would this ideal portal affect the relationships between its users?
14. To what extent would it encourage communication between different types of users?  Eg. Would this ideal web portal encourage researchers to collaborate with each other? Would the portal encourage clinicians to collaborate with each other? Patients to communicate with each other?
15. What would be the ideal attitudes that end users should adopt in order for the portal to work optimally?
16. Who else, besides patients, clinicians and researchers, should be considered in developing the web portal?
