# Supplementary material for: From code to care: Clinician and researcher perspectives on an optimal therapeutic web portal for acute myeloid leukemia
Source: PLoS One. 2024 Apr 18;19(4):e0302156. doi: 10.1371/journal.pone.0302156 (PMC11025855; doi:10.1371/journal.pone.0302156)
Supplement: S3 Appendix — (DOCX) [file pone.0302156.s003.docx]

**Supporting information: Codebook**

| **Name** | **Description** |
| --- | --- |
| Background information | This code covers all general information provided to participants regarding the research project or, alternatively, general information provided by them, but which is not relevant to our research objectives. |
| Confidentiality-privacy | This code includes all the excerpts that mention concerns about patient confidentiality (i.e., making sure that only authorized individuals have access to patients’ health information, especially potentially identifying information). It also covers mentions of privacy (i.e. the right of individuals to have control over how their personal information is collected, used and shared). |
| Consent process | This code covers all references to the patient consent process. |
| Data security | This code includes all excerpts that mention concerns about the logistics of preserving the confidentiality, integrity, and availability of data (for example, when sharing data). There is sometimes a tension between data security and portal usability. |
| Data-sharing | This code includes all excerpts that mention data-sharing (i.e., the practice of making data used for scholarly research available to other investigators) and the issues and concerns it may raise. |
| Device security | This code concerns any potential security issues associated with the device(s) that would be used by HCP to access the web portal (i.e., Is the device shared or private? Is it password protected? Is it connected to an intranet? etc.). |
| Device(s) used | This code concerns the type of device that would be used by the HCP to access the web portal (i.e., computer, tablet, phone). |
| Features | This code includes all the excerpts that mention ideal features that HCPs would like to see for the web portal. |
| Features\Clinical trial matching | This subcode includes all the excerpts that mention that the web portal could be used for clinical trial matching (i.e., to facilitate patient enrolment in clinical trials by identifying potential trials for interested patients and their HCPs). |
| Features\Collaboration | This subcode includes any excerpts that mention ways in which the web portal could contribute to collaboration between research teams, between researchers and clinicians, etc. |
| Features\Support services | This subcode includes all excerpts that mention support service features such as educational materials, FAQs, or staff who can answer questions or assist portal users. |
| Genetic knowledge | This code covers all excerpts that mention a concern about the genetic and genomic knowledge (or lack thereof) needed to navigate the web portal. In particular, there are excerpts where the researchers are concerned about the level of knowledge of clinicians and patients. |
| Information overload | This code includes all excerpts that mention a concern about information overload (i.e., too much information to navigate the portal efficiently). |
| Intellectual property | This code covers all passages that mention intellectual property issues associated with the portal. |
| Internet use | This code covers all passages that mention the use of the Internet or information technology in a very general way. |
| Knowledge of web portals | This code includes all references to use of existing web portals or websites. |
| Portal content | This code covers all mentions concerning the content of the portal. |
| Portal sustainability | This code includes all concerns about the maintenance and sustainability of the web portal, including human and financial resources, portal custodianship, etc. |
| Portal sustainability\Management | This subcode includes all excerpts that mention the responsibility for the management and maintenance of the web portal whether by an individual or an organization. |
| Portal sustainability\Resources | This subcode includes all references concerning human, material, or financial resources that would promote the success and sustainability of the web portal. |
| Portal uptake | This code includes all the excerpts that mention a barrier or a facilitator to the uptake of the web portal. |
| Portal uptake\Credibility | This subcode includes all the extracts that mention the credibility of the web portal, i.e. its ability to be a reliable source that provides trustworthy, unbiased and up-to-date information that inspires the trust of users. |
| Portal uptake\Searchability | This subcode refers to mentions where respondents address the structure of the database and the ability to search (i.e., by keyword, etc.) within it. |
| Portal uptake\Usability | This subcode includes all the excerpts that mention how easy the web portal is to use, including, for example, concerns about efficiency and navigability. |
| Portal uptake\Usefulness | This subcode includes all excerpts that mention ways in which the web portal could add value to existing information already available on the internet. |
| Portal uptake\Visibility | This subcode includes all excerpts that mention ways to make the web portal known to as many potential users as possible in order to promote uptake. |
| Socio-demographic | This code includes relevant sociodemographic information about respondents, such as their profession and the number of years of experience they have. |
| Users | This code includes all the extracts that mention concerns related to the users of the web portal. This is the question of who (i.e., researchers, clinicians, patients, etc.) should have access to what content (e.g., all content, restricted access to certain parts of the web portal, etc.). |
| Users\Clinicians | This subcode covers all passages that mention clinicians and other healthcare providers as users of the web portal. |
| Users\Other users | This subcode was created to include all excerpts mentioning other types of users than clinicians/researchers/patients (i.e., medical students, pharmaceutical companies). |
| Users\Patients | This subcode includes all references to potential patient access to the web portal and reflects the views of participants on the topic. |
| Users\Researchers | This subcode covers all passages that mention researchers as important end-users of the portal. |
| Web portal benefits | This code is about the perceived benefits of the web portal. The code is distinct from the subcode "usefulness". While usefulness is a prerequisite for the uptake of the web portal, benefits are more abstract and long-term outcomes. |
